# Role of exosomal microRNAs in cancer therapy and drug resistance mechanisms: focus on hepatocellular carcinoma

**DOI:** 10.3389/fonc.2022.940056

**Published:** 2022-07-15

**Authors:** Veronica Zelli, Chiara Compagnoni, Roberta Capelli, Alessandra Corrente, Mauro Di Vito Nolfi, Francesca Zazzeroni, Edoardo Alesse, Alessandra Tessitore

**Affiliations:** ^1^ Department of Biotechnological and Applied Clinical Sciences, University of L’Aquila, L’Aquila, Italy; ^2^ Center for Molecular Diagnostics and Advanced Therapies, University of L’Aquila, L’Aquila, Italy

**Keywords:** exosomes, miRNAs, hepatocellular carcinoma, therapy, drug resistance

## Abstract

Extracellular vesicles (EVs), defined as intercellular messengers that carry their cargos between cells, are involved in several physiological and pathological processes. These small membranous vesicles are released by most cells and contain biological molecules, including nucleic acids, proteins and lipids, which can modulate signaling pathways of nearby or distant recipient cells. Exosomes, one the most characterized classes of EVs, include, among others, microRNAs (miRNAs), small non-coding RNAs able to regulate the expression of several genes at post-transcriptional level. In cancer, exosomal miRNAs have been shown to influence tumor behavior and reshape tumor microenvironment. Furthermore, their possible involvement in drug resistance mechanisms has become evident in recent years. Hepatocellular carcinoma (HCC) is the major type of liver cancer, accounting for 75-85% of all liver tumors. Although the improvement in HCC treatment approaches, low therapeutic efficacy in patients with intermediate-advanced HCC is mainly related to the development of tumor metastases, high risk of recurrence and drug resistance. Exosomes have been shown to be involved in pathogenesis and progression of HCC, as well as in drug resistance, by regulating processes such as cell proliferation, epithelial-mesenchymal transition and immune response. Herein, we summarize the current knowledge about the involvement of exosomal miRNAs in HCC therapy, highlighting their role as modulators of therapeutic response, particularly chemotherapy and immunotherapy, as well as possible therapeutic tools.

## Introduction

Liver tumor is one of the most common types of cancers (1), displaying the 7^th^ highest age-adjusted incidence rate in the world. Hepatocellular carcinoma (HCC) is considered the most frequent liver neoplasm (1) and the third most common cause of cancer-related death worldwide (2), predominantly in Asian countries, due to late diagnosis and lack of effective surveillance programs for high-risk people. In this context, several non-invasive diagnostic biomarkers are considered, but, unfortunately, they do not reach necessary sensitivity and specificity, especially for early stage-HCC (3). On the other hand, liver biopsy is a limited procedure due to its invasiveness, and imaging methods (e.g. ultrasonography, magnetic resonance and/or computed tomography) are usually utilized for diagnosis, even though small tumors can be often missed (4, 5).

HCC initiation, development and progression are dependent both on intrinsic (e.g. gene mutations) and extrinsic (e.g. viral infections, western type diet intake, alcohol consumption) factors able to induce in liver cells the typical responses of malignant transformation, leading to apoptosis evasion, cell proliferation and survival, neovascularization (4). In particular, non-alcoholic fatty liver disease (NAFLD) is now considered the most important liver chronic disease (6) and it has been shown that, among HCC predisposing factors, not only high-fat (7), but also high-carbohydrate/western type diet can induce disease progression up to tumor formation in a NAFLD/NASH mouse model (8–10). The progression of the disease includes a passage through a cirrhotic stage in a large majority of HCC cases (up to 90%) (11). Oxidative damage (12), inflammation (13), hepatocyte compensatory regeneration (14), with consequent accumulation of gene mutations, are typical HCC features. Mutational HCC landscape includes many genes with different mutation frequency, such as *TP53* (30%), *CTNNB1/β-catenin* (26%), *ARID1A* (8%), *ARID2* (6%), *AXIN* (6%) (15). Several pathways, such TGF-β, Wnt/β-catenin, Hedgehog, Notch, EGF, HGF, VEFG, JAK/STAT, Hippo, and HIF are dysregulated and play a crucial role in HCC, leading to uncontrolled cell division and metastasis. For some of them, small promising molecules for therapeutic approaches are under investigation (16).

To date, the main approach in HCC management is radiofrequency ablation (RFA), surgical resection or liver transplantation, if feasible, and outcome of patients untreatable with resection curative methods is dependent on the response to the currently available therapies. Novel treatments, principally based on sorafenib and regorafenib, two tyrosine kinases inhibitors (TKIs) seem, however, to induce a moderate increase of HCC patients’ survival (17). MicroRNAs (miRNAs) are short non-coding RNA molecules able to regulate gene expression at the post-transcriptional level (18), playing a pivotal role in high-impact disorders, including neurodegenerative (19), cardiovascular (20) diseases and cancer (21, 22). They work by fine-tuning key physiological and pathophysiological processes, such as proliferation, survival, apoptosis, invasion, angiogenesis, epithelial-mesenchymal transition (EMT), metastasis and resistance to therapeutic treatments as well (23, 24). MiRNAs dysregulated expression levels are described both in tumor tissues and serum/plasma, where they are included in macromolecular protein complexes (25–27) or encapsulated in microvesicles/exosomes (28, 29) to be protected from endogenous RNase, being so easily detectable and quantifiable. Such properties allowed the identification of miRNAs as potential diagnostic, prognostic and predictive cancer biomarkers. Furthermore, aberrant miRNA expression in cancer led to the characterization of oncomiRs and tumor-suppressor miRs, playing a role in promoting or suppressing oncogenesis, respectively. The possibility to synthesize and obtain molecules able to specifically repristinate physiological conditions (i.e. antagomiRs inhibiting oncomiRs, mimics replacing tumor suppressor miRs) made these molecules of great interest for innovative cancer therapeutic strategies (30). Furthermore, several miRNAs were described as biomarkers for therapy response and disease-free survival/clinical progression in HCC patients (31–34).

In this review, we report recent advances on exosomal miRNAs in HCC, by focusing on their involvement and role in therapeutic responses.

## MiRNA biogenesis

In the canonical pathway, microRNAs are transcribed, from intergenic or intragenic genomic regions, by Polymerase II in the nucleus, thus originating long double stranded-hairpin primary transcripts (pri-miR). Subsequently, RNase III enzyme Drosha, associated to RNA binding protein (RBP) DGCR8 (DiGeorge critical region 8), cleaves pri-miRNAs to generate pre-miRNAs (60-100 nucleotides in length hairpin precursors) which are subsequently transferred to the cytoplasm by Exportin 5 through a Ran (Ras-related nuclear protein)-GTPase-dependent mechanism. There, pre-miRNAs are cleaved again by RNase enzyme Dicer, linked to the trans-activation-responsive RNA-binding protein (TRBP), to produce mature double-stranded miRNAs. Mature miRNAs associate to a member of Argonaute family (Ago1-2-3-4 paralogs in mammals) thus generating the ribonucleoprotein miRNA-induced silencing complex (miRISC). Two main mechanisms for miRNA-mediated regulation are described through the interaction between the seed region and specific partially or perfectly complementary microRNA responsive elements (MREs), mainly located at the level of target mRNA 3’-UTR, with consequent translation repression or mRNA decay by deadenylation followed by decapping, respectively (18, 35, 36). A DICER-independent mechanism has been described as well for pre-miR-451, involved in erythropoiesis. This is due to the stem-loop structure, too short to be processed by DICER. In this case, miRNA’s maturation requires direct loading into Ago2 and subsequent cleavage by its catalytic centre (37).

## Exosomes

Cells can secrete different types of extracellular vesicles (EVs). This feature is conserved from bacteria up to higher organisms (38, 39), and it was originally intended to discard unwanted or unnecessary molecules (40). However, it is known that EVs are involved also in exchanging nucleic acids, lipids and proteins among cells; moreover, they play a role in favouring intercellular communication, at the level of both physiological and pathophysiological processes (41). Three main types of EVs have been described: exosomes, microvesicles and apoptotic bodies which differ based on their biogenesis and release mechanisms, content, size and role (42, 43),.

**Figure 1:**
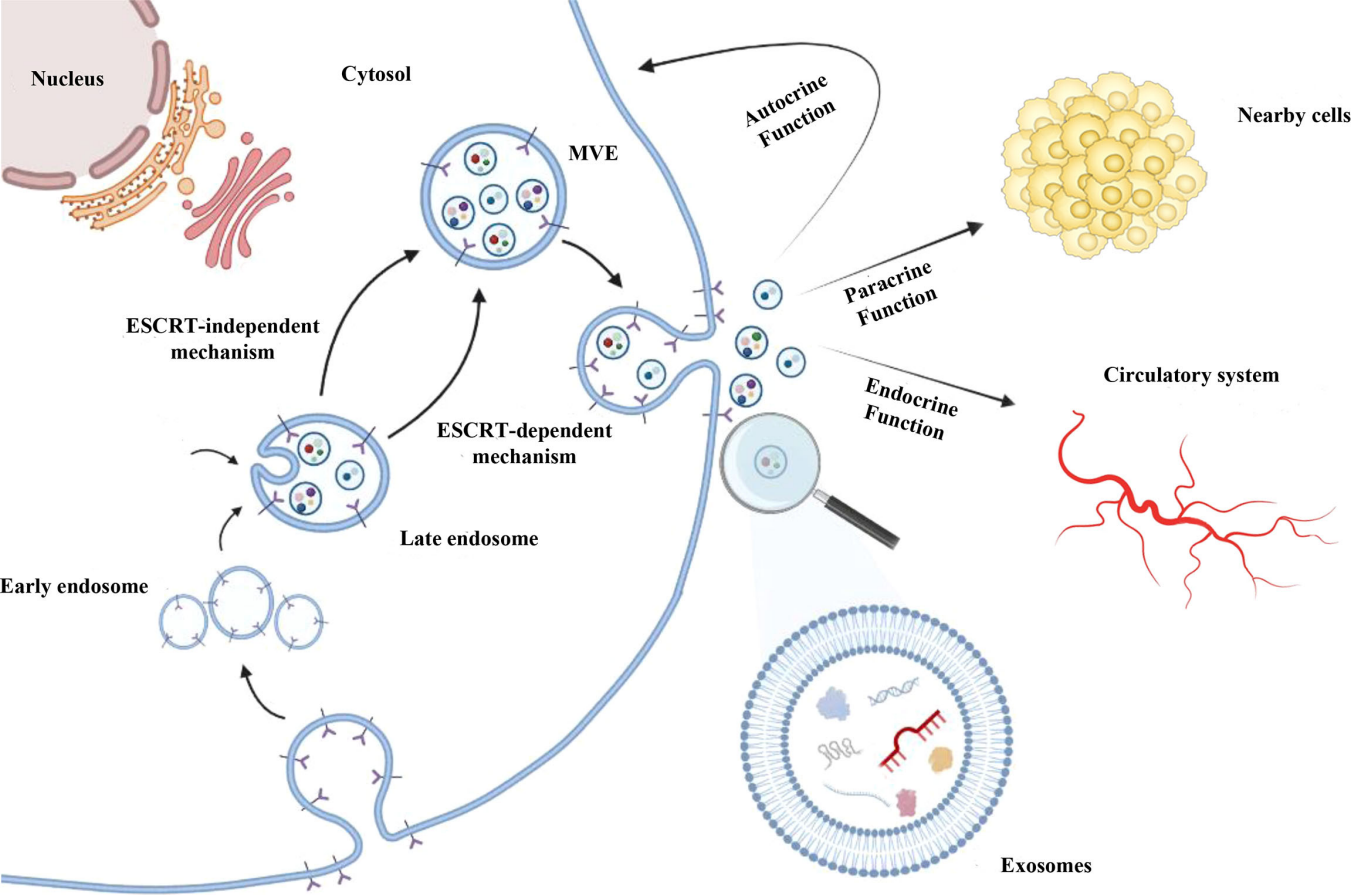
Exosomes biogenesis. After being generated by endocytosis at the level of endosomal system, exosomes are included as invagination within the multivesicular endosomes (MVEs) lumen and released as an outward budding upon the fusion of MVEs with the cell membrane. MVEs formation can occur mainly by endosomal sorting complex required for transport (ESCRT) pathway-dependent mechanism; ESCRT-independent mechanisms were also described. Exosomes can contain different cargos, such as proteins, lipids, DNA, mRNAs, lnRNAs and miRNAs. Exosomes are uptake by recipient cells thus favouring intercellular communication through their autocrine, paracrine and/or endocrine function.

Exosomes are nano-sized biovesicles (diameter 30-150 nm) secreted by all cell types. They can be detected in most of body fluids and are delimited by a lipid bilayer membrane which protects and aids to deliver cargos to recipient cells (44). Exosome biogenesis occurs as a part of membrane-trafficking processes: cargos are insourced and distributed into early endosomes at the level of endosomal system. Subsequently, late endosomes/multivesicular bodies (MVBs), containing intraluminal vesicles (ILVs), are generated from early endosomes. ILVs can sequester lipids, proteins and other cargos from cytosolic compartments and Golgi apparatus. After, MVBs containing cargos are driven at the level of the plasma membrane, where they merge with it, so that ILVs are released, as exosomes as an outward budding (43, 45, 46) (**Figure 1**).

The MVBs formation seems to be dependent also on stimulation by growth factors (47) and can occur mainly by endosomal sorting complex required for transport (ESCRT) pathway-dependent mechanisms (48). In addition, ESCRT-independent mechanisms have been described for MVBs formation (49–51) since it was demonstrated that MVBs can still form after depletion of ESCRT components (52). ESCRT (ESCRT-0/I/II/III) and accessory (e.g. Alix, TSG101, HSC70, HSP70) proteins are contained in exosomes following ESCRT-dependent MVEs generation, irrespective of the cell types, and for this reason they are considered as exosomal markers (42, 43, 53). On the other hand, in ESCRT-independent mechanism, other molecules (e.g. tetraspanins, CD63, CD9, CD81) are commonly found in exosomes, but also detected in other types of vesicles, such as MVs (54, 55). Based on information reported by some databases, exosomes can contain more than 8,000 proteins and 190 lipids. Integrins, tetraspanins, MHC-II complex proteins are described in the exosomal membrane, whereas other (CD55, trombospondin, ALIX, lactadherin) are included into exosomes during the biogenesis (56).

Exosome can load different and tissue-specific cargos, such as proteins, nucleic acids, lipids and metabolites, depending on the type of cell from which they are produced. Following their uptake by recipient cells, *via* exosomal fusion or endocytosis, they provide autocrine, paracrine and endocrine functions, thus favouring intercellular communications (55, 57) (**Figure 1**), participating in cell homeostasis (58), stimulating immune responses (59), promoting tissue repairing (60), cell survival (61), and modulating angiogenesis (62). For these reasons, they are considered as crucial modulators of intercellular cross-talks at the base of several high-impact diseases (63–65), including cancer.

## Exosomal miRNAs in HCC

Exosomal miRNAs have been shown to regulate tumor behavior and reshape tumor microenvironment (TME). Increasing evidence indicates that exosomal miRNAs contribute to HCC pathogenesis and progression by regulating processes such as cell proliferation, metastasis and immune response (66), thus showing considerable potential as diagnostic, predictive and prognostic biomarkers as well as new therapeutic tools (67). **Table 1** shows a comprehensive overview of exosomal miRNAs and related functions in HCC development and progression, based on the most recent literature. The utility of exosomes as vectors of biological therapeutic agents, such as miRNAs, has been actively explored in HCC (67). In the following sections, we will discuss, among those reported in **Table 1** the most relevant miRNAs described as mediators of therapeutic response and/or putative treatment tools in HCC.

**Table 1:** Overview of exosomal miRNAs involved in HCC pathogenesis.

	miRNA	Function in HCC	Anti-cancer drug(s)	Targets/pathway	Donor cell	Recipient cell	Reference
**Tumor Suppressor miRNAs**	miR-9-3p	Reducing proliferation and motility		HBGF-5, ERK1/2			(68)
	**miR-26a**	**Suppressing cell proliferation and migration**		**CCND2, CCNE2, CDK6**	**HEK293T**	**HCC cells (HepG2)**	(69)
	**miR-31 and miR-451a^1^**	**Anti-cancer activity**		**CDK2, SP1, BCL2α, MDR1**	**RCC**	**HCC cells (HepG2)**	(70)
	**miR-122**	**Increasing sensitivity to chemotherapeutic agents/inhibiting cell proliferation**	**5-fluorouracil (5-FU) and Sorafenib**	**ADAM10, IGF1R, CCNG1**	**AMSCs**	**HCC cells (HepG2)**	(71)
		Inhibiting growth and proliferation/increasing senescence		CAT1, FTF2B			(72)
	**miR-142 and miR-223**	**Inhibiting cell proliferation**		**STMN1, IGF-1R**	**TAMs**	**HCC cells (Huh7)**	(73)
	**miR-142-3p**	**Suppressing invasion and tumor growth**		**RAC1**	**TAMs**	**HCC cells (Hepa 1-6)**	(74)
	miR-145	Suppressing tumorigenesis and metastasis		GSK-3β/MMPs pathway			(75)
	miR-146a	Anti-cancer activity *via* immune system stimulation		M2 and T-cells			(76)
	miR-150-3p	Suppressing cancer progression		─			(77)
	**miR-199a-3p**	**Increasing sensitivity to anti-cancer drugs/inhibiting invasion**	**Doxorubicin**	**mTOR pathway**	**AMSCs**	**HCC cells (Huh7,SMMC-7721, PLC/PRF/5)**	(78)
			**Cisplatin (DDP)**	**ATM, mTOR and DNMT3A**	**HEK293T**	**HCC cells (Huh-7, Huh-7/DDP)**	(79)
	**miR-200b-3p**	**Suppressing angiogenesis**		**ERG**	**HCC cells (HLE, Hep3B)**	**Endothelial cells (Huvecs)**	(80)
	**miR-214**	**Reducing viability and invasion in combination with anti-cancer drugs**	**Oxaliplatin and Sorafenib**	**P-gp, SF3B3**	**hCEC**	**HCC cells (HepG2, Hep3B)**	(81)
	**miR-320a**	**Inhibiting proliferation, migration and metastasis**		**MAPK pathway (PBX3)**	**CAFs**	**HCC cells (MHCC97H and SMMC-7721)**	(82)
	**miR-335-5p**	**Inhibiting proliferation and invasion**		**CDC42, CDK2, CSNK1G2, EIF2C2, EIF5, LIMaK1, NRG1, PLK2, RGS19, TCF3, THBS1, YBX1, ZMYND8**	**CAFs**	**HCC cells (MHCC97L, MHCC97H, Huh7 and HepG2)**	(83)
	**miR-451a^1^**	Suppressing survival and angiogenesis		LPIN1			(84)
		**Suppressing drug resistance, proliferation, migration and invasion**	**Paclitaxel**	**ADAM10**	**HUC-MSCs**	**HCC cells (Hep3B and SMMC-7721)**	(85)
	**miR-490**	**Inhibiting metastasis**		**EGFR-AKT-ERK1/2 pathway (ERGIC3)**	**MCs**	**HCC cells (HepG2, Hep3B)**	(86)
	miR-718	Decreasing tumour aggressiveness and recurrence		HOXB8			(87)
	**miR-744**	**Inhibiting proliferation and chemoresistance**	**Sorafenib**	**PAX2**	**HCC cells (HepG2)**	**─**	(88)
**OncomiRs**	miR-10b	Promoting proliferation and metastasis		HIF-1α, HIF-2α			(89)
	miR-21			PTEN			
	**miR-21**	**Promoting angiogenesis, migration and tumorigenic function**		**PDK1/Akt pathway (PTEN)**	**HCC cells**	**HSCs**	(90)
				**─**	**HCC cells (SK-hep-1)**	**─**	(91)
	**miR-23a-3p**	**Regulating PD-L1 expression which helps tumor cells escape from antitumor immunity**		**PTEN**	**ER-stressed HCC cells**	**Macrophages**	(92)
	miR-25-5p	Enhancing cell motility		─			(93)
	**miR-32-5p**	**Promoting multidrug resistance**	**5-fluorouracil (5-FU), Oxaliplatin (OXA), Gemcitabine (GEM), Sorafenib**	**PI3K/Akt pathway (PTEN)**	**Multidrug-resistant HCC cells (Bel/5-FU)**	**sorafenib-sensitive HCC cells (Bel7402)**	(94)
	miR-92a-3p	Promoting EMT and metastasis		PTEN			(95)
	miR-92b	Enhancing migration ability of cells and decreasing NK cell-mediated cytotoxicity		CD69 on NK cells			(96)
	miR-93	Increasing proliferation and invasion		TP53INP1, TIMP2, CDKN1A			(97)
	miR-103	Increasing vascular permeability and metastasis		VE-Cad, p120, Zo-1			(98)
	**miR-135a-5p**	**Promoting survival, proliferation and chemotherapy resistance**	**Doxorubicin**	**VAMP2**	**HCC cells (HepG2)**	**─**	(99)
	**miR-155**	**Promoting angiogenesis**		**VEGF and HIF-1α, MVD**	**HCC cells (PLC/PRF/5** **and HuH7)**	**Endothelial cells (Huvecs)**	(100)
		Promoting proliferation		PTEN			(101)
	**miR-210**	**Promoting angiogenesis**		**SMAD4, STAT6**	**HCC cells (QGY-7703, HepG2, SK-Hep-1, Huh-7 and Hepa1-6)**	**Endothelial cells (Huvecs)**	(102)
	miR-224	Promoting proliferation and progression		glycine N-methyltransferase			(103)
	miR-655	Stimulating proliferation		MAPK/ERK pathway			(104)
	miR-1247-3p	CAFs activation and secretion of pro-inflammatory cytokines promoting cancer progression		NF-kB pathway (B4GALT3)			(105)
	miR-1273f	Stimulating proliferation and metastasis hypoxia-induced		Wnt/β-catenin pathway			(106)

AMSCs: Adipose tissue-derived mesenchymal stem cells; CAFs: Cancer-associated fibroblast; EMT: Epithelial-mesenchymal transition; hCEC:human cerebral endothelial cell; HEK293T: human embryonic kidney 293 cells; HSCs: Hepatocyte stellate cells; HUC-MSCs: Human umbilical cord mesenchymal stem cells; MCs: Mast cells; NK: Natural killer; RCC: renal carcinoma endothelial cells. TAMs: Tumor associated macrophages. ^1^The same miRNA was described in references 70 and 85.

MiRNAs described in sections 4.1 and 4.2 for their potential therapeutic applications are highlighted in bold.

### Exosomal miRNAs as modulators of therapeutic response

Several studies have described the ability of exosomal miRNAs to modulate HCC therapeutic response to different drugs, such as sorafenib, 5-fluorouracil (5-FU) and doxorubicin, or immunotherapy (107, 108). Experimental evidence suggests the involvement of exosomal miRNAs both in drug resistance mechanisms and in drug sensitivity improvement.

Lou et al. observed that miR-122-enriched exosomes, obtained from adipose tissue-derived mesenchymal stem cells (AMSC) after transfection with miR-122 expression plasmids, inhibited HCC cell proliferation and increased sensitivity to 5-FU and sorafenib both *in vitro* and in xenograft mouse models, by targeting and downregulating the expression of CCNG1, ADAM10 and IGF1R, genes involved in tumorigenesis and drug sensitivity in several cancer types. Thus, the authors highlighted the potential use of exosomal miR-122 to improve therapeutic response and revert drug resistance (71).

Similarly, exosomal miR-744 was able to rescue sorafenib sensitivity in resistant HepG2 cancer cells, by targeting PAX2, involved in the regulation of chemotherapy response in several cancers (88). Decreased level of miR-744 was found in HCC tissues, in exosomes from patients’ sera and HCC cells resistant to sorafenib: the restoration of miR-744 expression in HepG2 cancer cells and the subsequent release of miR-744-enriched exosomes led to decreased miR-744-induced cell proliferation and resistance to therapy (88).

On the contrary, miR-32-5p was found to contribute to multidrug resistance in HCC cells (94). The authors observed that exosomes from multidrug-resistant Bel/5-FU cells were able to deliver miR-32-5p into sensitive Bel7402 cells, thus inducing angiogenesis, EMT and drug resistance through PI3K/AKT pathway activation. Increased levels of exosomal miR-32-5p were also found in sera from HCC patients, associated with poor prognosis (94).

Resistance to chemotherapy, is one of the main factors responsible for high mortality rate in HCC patients, and the identification of mechanisms underlying chemoresistance as well as the enhancement of therapeutic options is of great clinical interest. In this context, the possible role of the exosomal tumor suppressor miR-199a-3p was recently explored in two studies (78, 79).

Lou et al. showed that exosome-mediated crosstalk between adipose tissue-derived mesenchymal stem cells (AMSCs) hyper-expressing miR-199a-3p and HCC cells increased the tumor sensitivity to doxorubicin by targeting mTOR signaling pathway. *In vivo* experiments, based on AMSC-Exo-199a injection into a HCC mouse model, confirmed increased doxorubicin anti-tumor effect in HCC (78).

Likewise, Zhang et al. observed that exo-miR-199a-3p restored sensitivity to cisplatin (DDP) and decreased tumor growth in chemo-resistant HCC cells. Due to the ability of exo-miR-199a-3p to overturn DDP resistance, the authors highlighted its great potential as an alternative therapeutic option in DDP-refractory HCC (79).

In a study aimed at elucidating the possible mechanism by which hepatitis B core antigen (HBc) promotes doxorubicin resistance in HCC, Wei et al. suggested that HBc led to upregulation of exosomal miR-135a-5p inducing cell proliferation, anti-apoptotic effects, and drug resistance. VAMP2 was identified as a novel miR-135a target, and its level decrease was linked to cell proliferation, apoptosis escape and drug resistance, thus identifying the miR-135a-5p/VAMP2 axis as a key regulatory chemo-resistance mechanism in HCC (99).

Exosomal miR-451a acts as tumor suppressor miRNA and its expression is down-regulated in HCC (84). Xu et al. used human umbilical cord mesenchymal stem cells (hucMSCs) derived exosomes to treat Hep3B cells and assess paclitaxel resistance. The authors demonstrated that exosomal miR-451 slowed EMT progression and reduced proliferation, migration and resistance to paclitaxel by suppressing ADAM10 in HCC cells, thus acting as a chemosensitivity-inducing factor and promoting HCC cell apoptosis (85).

Semaan et al. reported that the use of exosomal miR-214 from human cerebral endothelial cell-derived exosomes (hCEC-Exo-214) in combination with oxaliplatin or sorafenib could effectively reduce cancer cell viability and invasion of HepG2 and Hep3B cells compared to monotherapy. At the molecular level, this effect seems to be mediated by the glycoprotein P-gp and the splicing factor SF3B3 (81).

Inhibiting the release of exosomal oncomiRs, or increasing the effect of tumor suppressor miRNAs, could also play a synergic role in immunotherapy: for example, it has been shown that the inhibition of miR-23a-3p in HCC cancer cells could prevent their exosomal release and the consequent expression of PD-L1 in macrophages (92).

Similarly, it has been observed that high VEGF expression, in response to hypoxia, in TME can have immunosuppressive effect in tumors, resulting in decreased efficacy of PD-L1 and PD-1 inhibitor drugs (109, 110). Therefore, inhibition of VEGF and other hypoxia-induced factors, such as exosomal miRNAs involved in the regulation of angiogenesis in TME, could improve the efficacy of current immunotherapies (111). In this context, other studies led to hypothesize that targeting specific exosomal miRNAs, released from HCC cells and able to stimulate angiogenesis and HCC proliferation, such as miR-210 and miR-155, could therefore interfere with cellular crosstalk that promotes angiogenesis, further improving therapy (100, 102).

### Exosomal miRNAs as main therapeutic tools

Exosomes naturally act as carriers of nucleic acids, proteins and lipids from donor to recipient cells. They are characterized by high biocompatibility, low immunogenicity, low toxicity and ability to cross the blood-brain barrier. These features make them promising vehicles for the delivery of chemical and biological drugs (107). In this section, we focus on exosomal miRNAs described for their potential application as main biological therapeutic agents in HCC.

Zhang et al. observed that exosomes released from cancer-associated fibroblasts (CAFs) overexpressing miR-320a were able to transfer this miRNA into HCC cells and suppressed HCC cell proliferation and metastasis both *in vitro* and *in vivo* by targeting PBX3 (82).

Through the same experimental approach, Wang et al. demonstrated that CAFs-derived miR-335-5p-enriched exosomes could inhibit HCC cell proliferation and invasion, by regulating genes including CDC42, CDK2, CSNK1G2, EIF2C2, EIF5, LIMaK1, NRG1, PLK2, RGS19, TCF3, THBS1, YBX1, and ZMYND8 (83).

Xiong et al. showed that stimulation of mast cell with hepatitis C virus E2 envelope glycoprotein (HCV-E2) resulted in miR-490 expression increase in mast cells as well as in secreted exosomes. Furthermore, the delivery of Exo-miR-490 to HCC recipient cells inhibited the ERK1/2 pathway, thereby suppressing cell migration and metastasis (86).

MiR-21 is a well characterized oncomiR, involved in cell proliferation and metastases through the inhibition of genes such as PTEN (112), PDD4, RECK, and SULF-1 (113). The ability of miR-21 to confer resistance to chemotherapy in cancer cells has also been reported (112, 114).

Zhou et al. showed the ability of HCC-derived exosomal miR-21 to convert hepatocyte stellate cells (HSCs) to CAFs, resulting in angiogenesis promotion through increased secretion of VEGF, MMP2, MMP9, bFGF and TGF-β by CAFs. Results of this study provided further insight into the crosstalk between cancer cells and their microenvironment during tumor progression, providing new information of potential clinical utility in HCC (90).

Interestingly, Liang et al. used nanoparticles loaded with small interfering RNA (siRNA) to downregulate the expression of the pro-oncogenic factor Sphk2 in HCC cells to reduce exosomal miR-21, thus decreasing tumor cell migration and exosome-mediated tumorigenic function. The anti-tumor effect of Sphk2 siRNA was also demonstrated in a xenograft mouse model resulting in reduced HCC tumor progression. Therefore, targeting exosomal oncomiR secretion could represent a new therapeutic strategy (91).

Moh-Moh-Aung et al. reported that the downregulation of exosomal miR-200b-3p in HCC cells led to the promotion of angiogenesis through endothelial ERG expression increase, thus providing new insights into possible targetable mechanisms to improve the efficacy of anti-angiogenic therapies (80).

Immune cells play a key role in tumorigenic process, therefore the collection and possible engineering of exosomes from these cell types might represent an anti-tumor strategy that requires further investigation.

It has been shown that exosomal miR-142 and miR-223, transferred from tumor associated macrophages (TAMs) to HCC cells, can suppress cancer cell proliferation through the modulation of genes involved in cell cycle regulation, such as the miR-223 target gene STMN1 (73).

Furthermore, the use of the intravenous anaesthetic propofol, induced the secretion of miR-142-3p-enriched exosomes from TAMs, and the internalization of these vesicles into HCC cells led to the inhibition of cell invasion *in vitro* and tumor growth *in vivo* through down-regulation of miR-142-3p target gene RAC1 (74).

Liang et al. described an alternative approach to deliver antioncomiRs-enriched exosomes to HCC cells. In their study, HEK293T cells were engineered to secrete exosomes actively loaded with miR-26a by electroporation. These exosomes were able to selectively target HepG2 cells, thus decreasing cancer cell migration and proliferation *in vitro* though the inhibition of key cell cycle regulators such as CCND2, CCNE2, CDK6 (69).

The same approach was described by Pomatto et al. who used renal carcinoma endothelial instead of HEK293T cells, loaded with tumor suppressor miR-31 and miR-451a able to induce chemosensitivity (70). Overall, results of these two studies highlight the interesting possibility of using engineered exosomes as therapeutic agents.

## Conclusion

MiRNAs are considered pivotal modulators of intercellular crosstalk and miRNA transfer *via* exosomes has been described as one of the possible strategies by which resistant HCC cells can share their resistance with neighbouring cells, thus hindering therapies.

In this context, based on the results principally obtained from *in vitro* and *in vivo* models, exosomes can be also considered as promising vehicles of miRNAs for therapeutic purposes, representing a great resource for the design of new treatment strategies with potential efficacy, especially in combination with chemotherapy, TKIs or immunotherapy, currently considered as a cutting-edge cancer treatment.

In addition to exosomal miRNAs directly targeting HCC cells, an interesting alternative could also be HCC tumor microenvironment targeting, in terms of CAFs, immune cells or tumor endothelial cells, on which exosomal miRNAs could induce desirable responses.

Furthermore, technological advances, focused for example on vesicles and donor cells engineering, offer an unprecedented opportunity to improve and provide novel tools for potential therapeutic applications of exosomal miRNAs in HCC and other types of cancers.

Overall, although more in-depth studies to elucidate the exact biological role and possible applicability of exosomal miRNAs in HCC treatment are required, these mediators can represent promising factors of potential therapeutic utility in HCC patients.

## Author contributions

VZ, CC, and AT: original draft preparation; VZ, CC, RC, and AC: Bibliographic information; MDVN and FZ: reading and editing manuscript; EA and AT: conceptualization, review and editing manuscript. All authors contributed to the article and approved the submitted version.

## Conflict of interest

The authors declare that the research was conducted in the absence of any commercial or financial relationships that could be construed as a potential conflict of interest.

## Publisher’s note

All claims expressed in this article are solely those of the authors and do not necessarily represent those of their affiliated organizations, or those of the publisher, the editors and the reviewers. Any product that may be evaluated in this article, or claim that may be made by its manufacturer, is not guaranteed or endorsed by the publisher.
